# Macroevolutionary drivers of morphological disparity in the avian quadrate

**DOI:** 10.1098/rspb.2023.2250

**Published:** 2024-02-21

**Authors:** Pei-Chen Kuo, Guillermo Navalón, Roger B. J. Benson, Daniel J. Field

**Affiliations:** ^1^ Department of Earth Sciences, University of Cambridge, Downing St, Cambridge CB2 3EQ, UK; ^2^ Department of Earth Sciences, University of Oxford, 3 S Parks Rd, Oxford OX1 3AN, UK; ^3^ American Museum of Natural History, 200 Central Park West, New York, NY, USA; ^4^ Museum of Zoology, University of Cambridge, Downing St, Cambridge CB2 3EJ, UK; ^5^ Fossil Reptiles, Amphibians and Birds Section, Natural History Museum, Cromwell Road, London SW7 5BD, UK

**Keywords:** birds, quadrate, morphology, integration, many-to-one mapping, one-to-many mapping

## Abstract

In birds, the quadrate connects the mandible and skull, and plays an important role in cranial kinesis. Avian quadrate morphology may therefore be assumed to have been influenced by selective pressures related to feeding ecology, yet large-scale variation in quadrate morphology and its potential relationship with ecology have never been quantitatively investigated. Here, we used geometric morphometrics and phylogenetic comparative methods to quantify morphological variation of the quadrate and its relationship with key ecological features across a wide phylogenetic sample. We found non-significant associations between quadrate shape and feeding ecology across different scales of phylogenetic comparison; indeed, allometry and phylogeny exhibit stronger relationships with quadrate shape than ecological features. We show that similar quadrate shapes are associated with widely varying dietary ecologies (one-to-many mapping), while divergent quadrate shapes are associated with similar dietary ecologies (many-to-one mapping). Moreover, we show that the avian quadrate evolves as an integrated unit and exhibits strong associations with the morphologies of neighbouring bones. Our results collectively illustrate that quadrate shape has evolved jointly with other elements of the avian kinetic system, with the major crown bird lineages exploring alternative quadrate morphologies, highlighting the potential diagnostic value of quadrate morphology in investigations of bird systematics.

## 1. Introduction

Crown birds (Neornithes) are the most species-rich group of extant tetrapods, showcasing an extraordinary diversity of body forms and ecological disparity across virtually all major subaerial environments [1,2]. The origin of much of this diversity is thought to lie in a large-scale adaptive radiation in the aftermath of the Cretaceous-Palaeogene (K-Pg) mass extinction (e.g. [3]). This episode of diversification rapidly gave rise to ecologically disparate avian lineages [4] and was characterized by the rapid accumulation of morphological disparity across the skeleton (e.g. [5]), including striking variability in the morphology and ecological specialization of the feeding apparatus (e.g. [6,7]). Recent research has challenged assumptions regarding straightforward relationships between dietary traits and large-scale macroevolutionary patterns of craniofacial shape variation (e.g. beak shape [8]; cranial shape [9]; but see [10]) although more nuanced relationships may exist at more restricted phylogenetic scales (e.g. in anseriforms [11] and charadriiforms [12]; although see [13]) and/or with finer anatomical traits within specific regions of the head [14].

**Figure 1.**
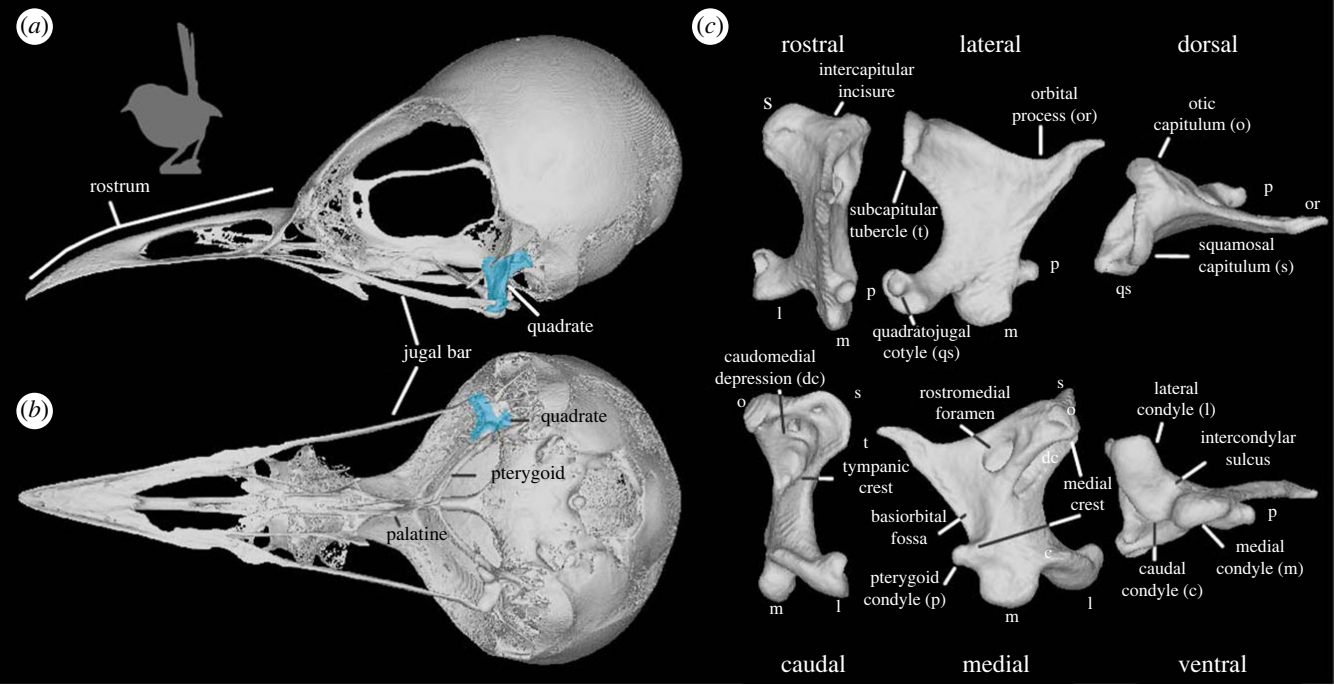
Quadrate anatomy in Neornithes. Skull of *Malurus melanocephalus* (UMMZ 224775), the species with the quadrate closest to the mean shape in our dataset in lateral (*a*) and ventral view (*b*), and its quadrate in rostral, lateral dorsal, caudal, medial and ventral views (*c*). Quadrate of *Malurus melanocephalus* > highlighted in blue in (*a*) and (*b*).

Despite the biomechanical importance of the palate system for feeding in neornithine birds (e.g. [6,15]), detailed investigations of the factors underlying the evolution of avian palatal disparity are scarce (although see [16] exploring allometry of the vomer). Within this system, the quadrate bone (*os quadratum* [17], hereafter ‘quadrate’) serves a key role in feeding biomechanics and cranial kinesis as it articulates directly with numerous components of the feeding apparatus: the mandible, the bony palate via the pterygoid bone, and the rostrum via the quadratojugal's connection with the jugal bars [18–21] (figure 1). During jaw closure, the quadrate is pulled rostrally by the action of the pterygoideus muscle group and transfers adduction forces through two sets of pushrods (the pterygoid-palatine complex and the jugal bars) to the mandible and rostrum which ultimately causes dorsiflexion of the beak at kinetic positions situated at its base and/or at its tip [22]. Furthermore, the quadrate provides attachment sites for some key muscles, such as *M. protractor pterygoidei et quadrati*, *M. pseudotemporalis profundus* and *M. adductor mandibulae caudalis*, which are involved in the movement of both the upper and lower jaws [19]. Different neornithine lineages have modified this basic configuration into a plethora of disparate morphologies thought to be tuned to functions and behaviours related to procuring different types of food [6]. As such, the considerable morphological variation exhibited by avian quadrates may reflect this ecological and functional variation. However, since the quadrate is part of a functionally integrated kinetic system involving several interconnected skeletal components, macroevolutionary patterns in quadrate geometric disparity may also reflect constraints imposed by the morphological evolution of its neighbouring bones.

Here, we quantitatively explore macroevolutionary links between the morphology of the avian quadrate and feeding, foraging and habitat ecology across a wide range of extant birds, while taking into consideration functional and developmental relationships of this crucial skeletal element with other osteological components of the palate and skull. Our work clarifies the extent to which adaptation for feeding function has driven morphological disparity of a key component of the avian feeding apparatus and highlights the influence of structural constraints and morphological integration on dictating skeletal geometry.

## 2. Material and methods

### (a) Dataset, phylogenetic hypothesis and ecological information

**Figure 2.**
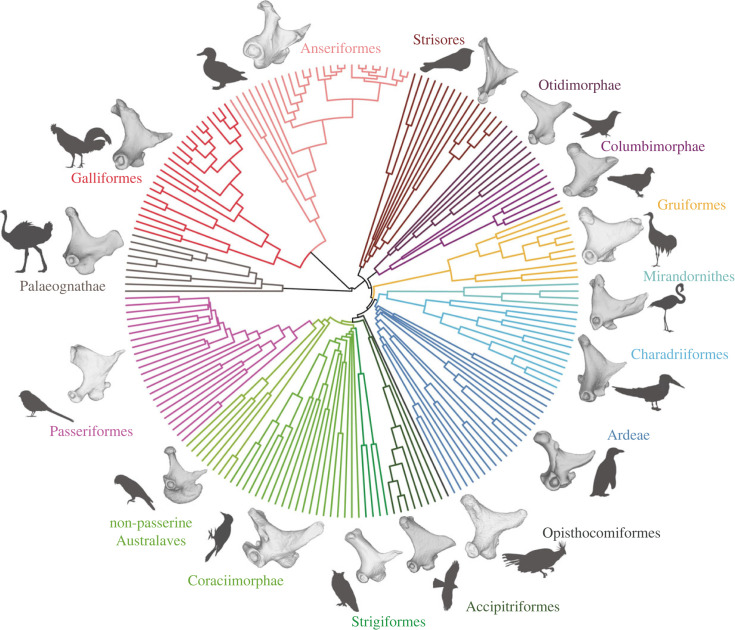
Quadrate geometric disparity in Neornithes. Time-calibrated phylogeny of the taxa included in this study (*n* = 200) including exemplar quadrate morphologies for each clade. Palaeognathae (*Struthio camelus* UMZC uncatalogued), Galliformes (*Alectura lathami* NHMUK S/2010.1.31), Anseriformes (*Anas aucklandica* NHMUK S 2006.39.1), Strisores (*Eurostopodus mysticalis* NHMUK S 1981.95.6), Otidimorphae (*Hierococcyx fugax* FMNH 357420), Columbimorphae (*Treron capellei* UMMZ 220454), Gruiformes (*Balearica pavonina* NHMUK 1859.10.26), Mirandornithes (*Phoenicopterus roseus* UMZC 346.B), Charadriiformes (*Rynchops niger* FMNH 376309), Ardeae (*Spheniscus humboldti* NHMUK S 2000.7), Opisthocomiformes (*Opisthocomus hoazin* NHMUK 1961.6.1), Accipitriformes (*Accipiter nisus* NHMUK S 1982.149.1), Strigiformes (*Tyto alba* NHMUK S 1899.22.1), Coraciimorphae (*Picus viridis* NHMUK S 1982.5.1), non-passerine Australaves (*Nestor notabilis* FMNH 23530) and Passeriformes (*Malurus melanocephalus* UMMZ 224775).

Our dataset includes 200 bird species and samples most major neornithine subclades, generally following the higher-level sampling of Prum *et al*. [4] (figure 2; electronic supplementary material). A time-calibrated phylogeny of these 200 species was constructed using the backbone of a recent fossil-calibrated genome-level molecular analysis [4] and several other subclade-focused studies for the interrelationships within the following neornithine clades: Tinamiformes [23], Galliformes [24], Anseriformes [25], Apodiformes [26], Rallidae [27], Podicipedidae [28], Accipitridae [29], Psittaciformes [30] and Passeriformes [31]. Our topology was constructed using Mesquite v.3.61 [32], and the resulting tree was temporally calibrated using *paleotree* v.3.4.4 [33] within the *R* statistical environment v.4.2.1 [34]. Downstream analyses were also undertaken in *R*. We sourced divergence time estimates from recent genome-level molecular analyses [4,31], and the ages of remaining uncalibrated nodes lacking recent molecular divergence time estimates were estimated using the ‘timePaleoPhy’ function of *paleotree*, using the ‘equal’ time-scaling method.

Feeding and foraging autecology of each species in our dataset was characterized using eight classes of ecological information: trophic level (discrete: herbivore, carnivore, scavenger), trophic niche (discrete: frugivore, granivore, nectarivore, terrestrial herbivore, aquatic herbivore, invertivore, vertivore, aquatic predator, scavenger), habitat (discrete: forest, woodland, riverine, grassland, wetland, shrubland, human modified, marine, coastal and rock), primary foraging lifestyle (discrete: aerial, insessorial, terrestrial, aquatic and generalist) and main diet (two categories: diet and diet.50). Diet was treated as discrete following the EltonTraits 1.0 database [35] and was divided into five categories: fruinect, invertebrate, omnivore, plantseed and vertfishscav. Diet.50 was also treated as discrete (fruit, invertebrate, nect, omnivore, planto, plantseed, seed, vert.ect, vert.end, vert.fish and vertfishscav). The Diet.50 variable is based on the semi-quantitative dietary preference data from the EltonTraits 1.0 database and these semi-quantitative dietary preferences were composed of 10 different dietary categories coded as 10 binned increments representing the importance of each item in the diet of each species. Main diet (Diet.50) follows the majority dietary preference (greater than 50%); for taxa whose majority dietary preference is lower than 50%, the first two major dietary preferences were chosen. Additional variables examined were quadrate size (continuous, quantified as log_10_-transformed centroid size, see below), semi-quantitative dietary preferences (binned in 10 increments, see above) and use of the beak during feeding (discrete; cracking/ripping, tearing, pecking/grazing, grabbing/gleaning, probing, filtering). Variation by taxonomic group was also assessed. Data on trophic level, trophic niche, habitat and primary foraging lifestyle were retrieved from the global AVONET database [36], and the size of the quadrate was estimated from the centroid size of landmark constellations as one of the outputs of the ‘gpagen’ function of the *geomorph* 4.04 *R* package [37]. Semi-quantitative dietary preferences for each species were obtained from the global EltonTraits 1.0 database, and this noncontinuous matrix was transformed into a Euclidean distance matrix using the function ‘dist’ from the *R* package *stats* v.3.6.2 [34], which we then transformed using a principal coordinates analysis (PCoA). The scores from this PCoA were then used as variables (diet.matrix) for downstream analyses. To code the use of the beak during feeding (UBF), we followed the methods described in [8].

### (b) Quadrate shape

We captured the complex morphology of the quadrate bone from each species by means of landmark-based geometric morphometrics applied to three-dimensional surfaces extracted from micro-computed tomography (CT) scanned museum specimens. Specimens came from three sources: (i) CT scans of *Ptilopachus petrosus* were obtained from the Natural History Museum, Tring, (ii) numerous specimens housed in the University of Cambridge Museum of Zoology were scanned specifically for this study and (iii) remaining scans from several collections around the world were sourced from the project TEMPO Birds, managed by co-author R.B.J.B., downloaded from the online repository MorphoSource (https://www.morphosource.org/; electronic supplementary material). Three-dimensional volumes were generated from VGSTUDIO MAX 3.5.4 (Volume Graphics) and Avizo 2019.3 (Thermo Fisher Scientific). After segmenting each quadrate, internal voids were filled in Avizo to provide a solid surface and prevent artefacts during the sliding treatment of curve and surface semi-landmarks. Finally, three-dimensional volumes were transformed as three-dimensional surfaces for landmarking in Avizo. Our landmarking scheme is a modified version of [38] and was focused on characterizing: (i) the articular surfaces between the quadrate and its neighbouring bones, (ii) the position of muscle attachments and (iii) the overall shape of the quadrate (a detailed description of our landmarking scheme, including anatomical criteria used to place our shape coordinates, is presented in the electronic supplementary material). Series of curve semi-landmarks and patches of surface semi-landmarks were initially positioned using arbitrary numbers of points for each specimen, and then resampled to equal counts of evenly spaced semi-landmarks before downstream analysis using the ‘digit.curves’ function of *geomorph* (see [39]; for further details see electronic supplementary material). The final densely sampled landmark configurations (eight landmarks, 509 curve semi-landmarks and 797 surface semi-landmarks) were subjected to a generalized Procrustes analysis to remove geometric information related to scaling, translation and rotation using the ‘gpagen’ function in *geomorph*. The Minimum Bending Energy sliding method [39,40] was used to slide the majority of semi-landmark coordinates (see electronic supplementary material for details about digitizing surface semi-landmarks and generalized Procrustes analysis).

To address potential statistical biases introduced by our dense landmarking scheme (e.g. [41]), we tested the equivalence between our original shape data and a subsampled version which only included discrete landmarks and curve semi-landmarks. For this, we calculated Procrustes distances (PD) between all pairs of species for both datasets, and performed a Mantel test between both distance matrices using the ‘mantel’ function from the *R* package *vegan* v.2.6-2 [42], using Pearson's correlation and 9999 permutations to test for statistically significant differences among sets. Our results show that both distance matrices are strongly and significantly correlated (*r* = 0.9511; *p* < 1×10^–4^). Visual exploration of the differences among PD between datasets (electronic supplementary material, figure S25 A) revealed that PD seem to be lower overall in our full dataset (PD_full_) compared with those in the subsampled version (PD_sub_), suggesting that following denser landmarking schemes may lead to less marked estimated differences among morphologies, in line with expectations. Finally, we explored whether differences in PD between these datasets disproportionately affect certain morphologies/species. For that, we applied the *R* ‘heatmap’ function to plot differences between PD among pairs (Δ: PD_full_ – PD_sub_). Our results showed no clear patterns (electronic supplementary material, figure S25 B), indicating that the slight differences in PD found between the two datasets do not disproportionally affect certain morphologies, and therefore, including our patches of surface semi-landmarks do not lead to significant biases in resultant shape estimates.

### (c) Principal components analysis

Shape data (Procrustes coordinates) were subjected to a principal components analysis (PCA) to visualize quadrate shape variation using the ‘gm.prcomp’ function in *geomorph*. Shape changes associated with each of the first five major axes of total quadrate shape variation (PC1–PC5) were displayed as deformations warped on the three-dimensional surface of the quadrate of the individual species closest to the mean quadrate shape in our sample, which was Red-backed Fairywren, *Malurus melanocephalus*. Specifically, this three-dimensional surface and the mean shape from the sample were projected onto the scores representing the 0.05 and 0.95 quantiles for each PC axis by means of thin-plate spline deformation [43] using the function ‘tps3d’ from the package *Morpho* v.2.10 [44] and ‘shape.predictor’ from *geomorph*. We also plotted the respective landmark configurations onto the deformed meshes using ‘shape.predictor’, and coloured landmark constellations according to per-landmark-variances using the ‘hot.dots’ function (freely available following this link: https://zenodo.org/record/3929193).

### (d) Multivariate statistics

We ran phylogenetic generalized least-squares (PGLS) linear models of differing complexity (see electronic supplementary material: Additional results—PGLS) to evaluate the main effects and interactions of all the ecological variables, size (log_10_ centroid size) and phylogenetic group with respect to quadrate shape variance using the ‘procD.pgls’ function in *geomorph*. Since this function assumes a single-rate Brownian motion model of evolution [37], we re-scaled the branches of our calibrated phylogenetic tree to adjust the phylogenetic-variation-covariation matrix to a Brownian motion process (e.g. [45]). For this, we ran each PGLS regression once and then estimated the value of Pagel's lambda in the residuals from the PGLS regression using the ‘phylosig’ function from the package *phytools* v.1.03 [46] (for all values of lambda see electronic supplementary material). Then, we used this value of Pagel's lambda to rescale our phylogeny using the function ‘rescale’ in the package *geiger* v.2.0.10 [47] and ran each PGLS regression a second time.

Finally, we explored shape covariation between quadrate geometry and the shapes of neighbouring bones (i.e. evolutionary integration; see electronic supplementary material, tables S5–S8 and figure 5 for the full list of relationships tested). To do so, we obtained shape data for the pterygoid-palatine complex (PPC, vomer excluded), jugal, cranium, rostrum and mandible from [48] and pruned our dataset to match the species list from that study. The pruned quadrate dataset (*n* = 97) was subjected to Phylogenetic Two Blocks Partial Least-Squares (p-2BPLS) using the function ‘phylo.integration’ in *geomorph* and a rescaled phylogeny using the lambda value estimated from shape residuals from the simplest PGLS linear model (shape∼1) to comply with the assumption of Brownian motion from p-2BPLS*.* We conducted p-2BPLS among a number of variables, including: (i) shape (Procrustes coordinates) of the quadrate and the shape of each bone with which it articulates, (ii) shape of the individual regions of the quadrate (i.e. internal integration) and (iii) shape of the individual regions of the quadrate and the shape of each bone with which the quadrate articulates. The full list of variables explored is available in the electronic supplementary material. Because different relationships among morphology, ecology and integration can emerge at different phylogenetic scales [8,9], we repeated all the above-mentioned analyses across Neornithes as a whole, as well as subsets corresponding to Telluraves (landbirds) and non-Telluraves. We used the function ‘compare.pls’ from *geomorph* to test for statistical differences in the strength of integration (i.e. z-scores from the p-2BPLS) between the same bone associations among the three clades.

## 3. Results

### (a) Main patterns of quadrate shape variation in Neornithes

The first five principal components (PC) account for only 59.82% of total avian quadrate shape variance (PC1: 28.30%; PC2: 11.64%; PC3: 7.66%; PC4: 6.54%; PC5: 5.48%; see electronic supplementary material, figure S12 and figure S16 for details of shape patterns associated with each major axis), and each subsequent PC explains less than 5% of total shape variance.

**Figure 3.**
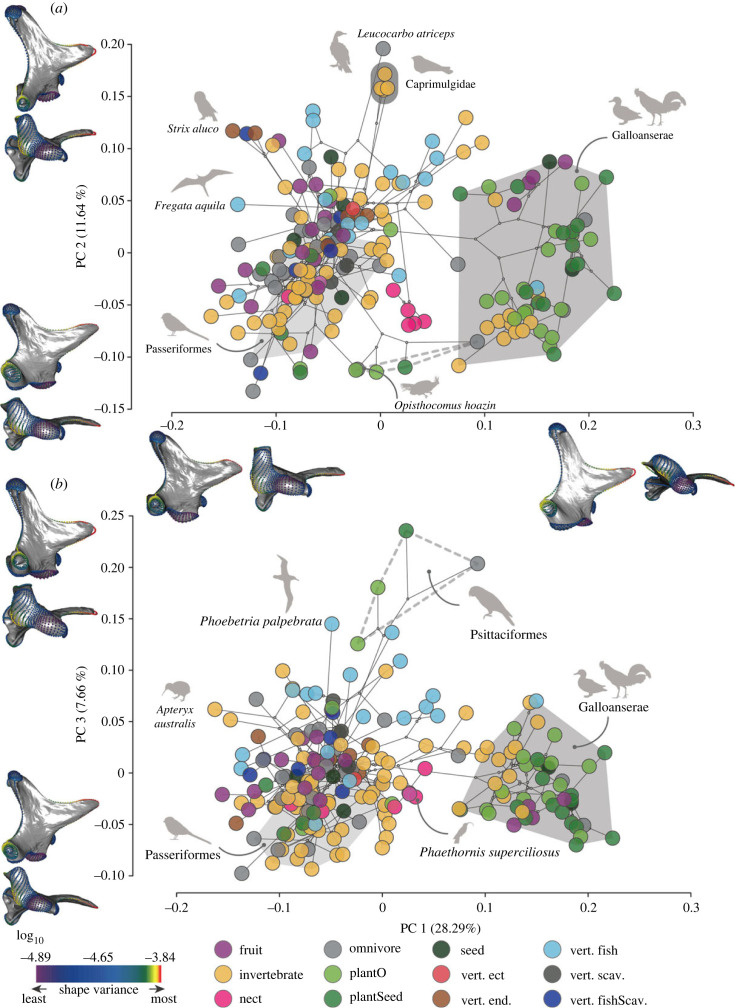
Macroevolutionary patterns of quadrate shape variation and dietary ecology in Neornithes. Phylomorphospace of the first three axes of quadrate shape variation. (*a*) PC1-PC2 and (*b*) PC1-PC3. Quadrate models are shown in lateral and ventral views and represent 0.95 quantiles of shape variance along PC2 and PC3 (top) in (*a*) and (*b*), respectively; 0.05 quantiles of shape variance along PC2 and PC3 (bottom) in (*a*) and (*b*), respectively; 0.95 quantiles of shape variance along PC1 (right) in (*a*) and 0.05 quantiles of shape variance along PC1 (left) in (*a*). Landmarks and semi-landmarks on the quadrate models are coloured according to log10-transformed per-landmark Procrustes variance. Bird species are labelled by main dietary category (see Material and methods). Convex hulls represent the approximate area of the morphospace in (*a*) and (*b*) occupied by Galloanserae (dark grey), Passeriformes (light grey) and Psittaciformes (dashed grey line). Individual taxa and family-level clades are labelled.

PC1 separates Galloanserae (Galliformes + Anseriformes, with positive scores) from other Neornithes (mostly with negative scores, see the dark grey convex hull in figure 3; electronic supplementary material, figure S13 for a version of this figure coloured by clade). This PC axis describes important differences in the otic process, the quadratojugal cotyle, the pterygoid condyle, the mandibular process and the quadrate body (figures 1 and 3; electronic supplementary material, figure S16). Quadrates associated with a narrow otic process (i.e. with the two otic capitula positioned close to each other), a narrow mandibular process (i.e. the medial and lateral condyle rostrocaudally aligned), and a slender quadrate body, such as those of Galloanserae, occupy the positive side of PC1 (figure 3; electronic supplementary material, figure S13A–D, PC1 positive). On the other hand, quadrates with negative scores on PC1 display a wider otic process (otic capitula relatively widely separated), a laterally positioned quadratojugal cotyle, a rostrally oriented pterygoid condyle, a caudally expanded mandibular process (i.e. caudal condyle well developed) and a wider quadrate body (figure 3; electronic supplementary material, figure S16, PC1 negative).

PC2 captures shape variance associated with muscle attachments and articular surfaces (figure 3; electronic supplementary material, figure S16, PC2). Taxa with curved quadrate bodies, curved lateral and medial crests, and widely spaced capitula on the otic process exhibit positive scores along PC2 (figure 3; electronic supplementary material, figure S16, PC2 positive). Such quadrates also exhibit a lateromedially curved articular surface of the squamosal capitulum with an ovoid outline and a medially facing otic capitulum due to a markedly curved medial crest (best typified in Strigiformes; figure 3*a*; electronic supplementary material, figure S13A,E–G). These quadrate shapes are also characterized by relatively short and robust orbital processes directed lateroventrally, and ventrally facing quadratojugal cotylae with relatively small articular fossae (electronic supplementary material, figure S16, PC2 positive), as seen in the Imperial Cormorant (*Leucocarbo atriceps*) and nightjars (Caprimulgidae), and mandibular processes with dorsally tilted caudal condyles divided by a deep groove (intercondylar sulcus; figure 3*a*; electronic supplementary material, figure S13A,E–G). By contrast, quadrates exhibiting negative PC2 scores are associated with narrowly spaced capitula on the otic process (figure 3*a*; electronic supplementary material, figure S16, PC2 negative), as in some Anseriformes (figure 3*a*; electronic supplementary material, figure S13A,E–G), and a squamosal capitulum with a relatively flat, rounded articular surface (electronic supplementary material, figure S16, PC2 negative). Such quadrates also have dorsomedially facing orbital processes with pointed tips and high aspect ratios, and mandibular processes in which the intercondylar vallecula and the caudal condyles are weakly developed, as in some parrots (Psittaciformes; dashed line in figure 3) and the Hoatzin (*Opisthocomus hoazin*; figure 3*a*; electronic supplementary material, figure S13A,E–G).

PC3 corresponds to shape differences in the mandibular process between parrots and all other avian lineages (figure 3*b*; electronic supplementary material, figure S13, B, E, H–I). The parrot quadrate is mainly characterized by: (i) a mandibular process adjacent to the pterygoid condyle and significantly separated from the quadratojugal cotyle, (ii) a short but robust orbital process with a blunt tip, (iii) a rostrally displaced medial condyle (or rostral portion of the mandibular process), (iv) a dorsally displaced caudal condyle (or caudal portion of the mandibular process) and (v) a caudomedially displaced lateral condyle (or lateral part of the mandibular process), which places the three main condyles in rostrocaudal alignment (figure 3*b*; electronic supplementary material, figure S16, PC3 positive). This configuration differs from the more common condition in which quadrates bear a longer orbital process with a pointed tip and the mandibular process is widely separated from the pterygoid condyle but close to the quadratojugal cotyle, with three clear condyles forming a triangular configuration in ventral view, such as in Passeriformes (figure 3*b*; electronic supplementary material, figure S16, PC3 negative). Shape changes associated with the remaining main axes of shape variation are detailed in the electronic supplementary material.

### (b) Quadrate shape and species autecology

**Figure 4.**
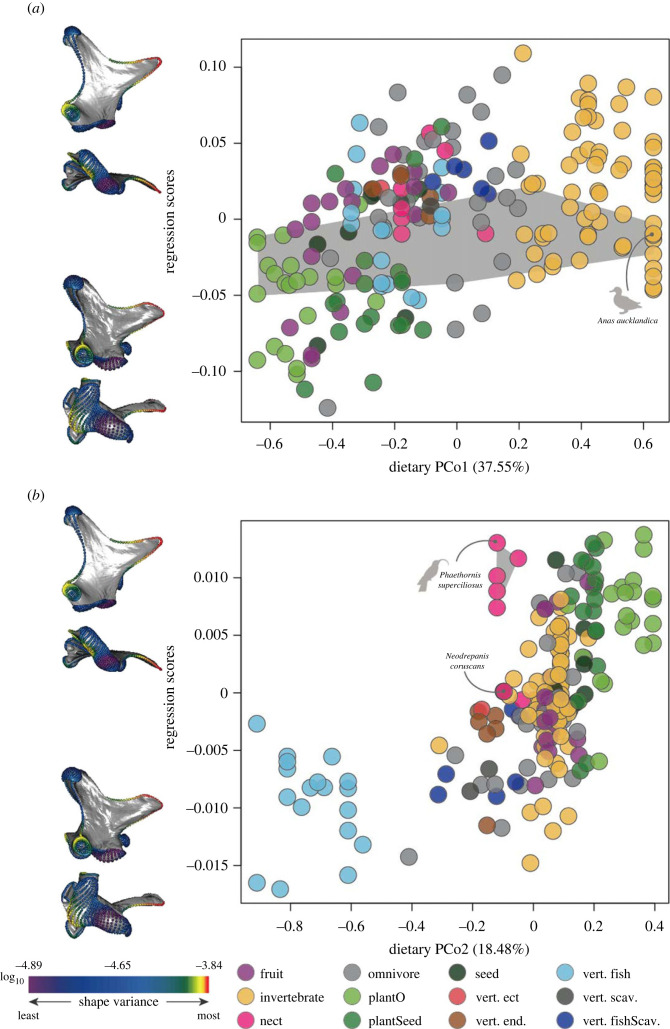
The evolutionary relationship between quadrate shape and the two main axes of dietary variation in Neornithes. Relationship between quadrate shape (regression scores (shape variance∼Diet matrix)) and scores from the first two PCo axes (PCo1(A) and PCo2(B)) of semi-quantitative dietary variation (see Methods). Bird species are labelled by their main dietary category (see Methods). Quadrate models represent the 0.95 and 0.05 quantiles of shape variance along the regression vector that is maximally associated with each of the major axes of shape variation. Quadrates are shown in lateral and ventral views. Landmarks and semi-landmarks on the quadrate models are coloured by log10-transformed per-landmark Procrustes variance. Shaded area in *a* indicates the variance exhibited by Anseriformes; shaded area in (*b*) indicates the variance displayed by Trochilidae.

Our PGLS regressions illustrate that all ecological factors investigated (i.e. trophic level, diet, beak use during feeding, primary habitat and primary lifestyle) exhibit non-significant correlations with quadrate shape (table 1), including when corrected for the effects of other potential explanatory factors such as the main effects of phylogenetic group and allometry, and group-specific allometries (electronic supplementary material, table S2). Similar quadrate shapes are associated with widely varying dietary ecologies (i.e. one-to-many mapping of form to ecology [8]) and very different quadrate shapes are associated with similar dietary ecologies (i.e. many-to-one mapping of form to ecology; figure 4).

**Table 1.** Summary of PGLS linear models for Procrustes coordinates (quadrate shape) in Neornithes (*n* = 200) as a function of ecological information, and interactions. Numbers in italics indicate statistical significance, * for *p* value < 0.05 and ** for *p* value < 0.01*.*

ANOVA syntax	*λ*	SS	MS	*R^2^*	*F*	Z	*p-*value
shape∼log10(centroid size)	*0.8108873*	*0.002155*	*0.00215464*	*0.01583*	*3.1838*	*2.7303*	0.0022 **
shape∼Phy.group	*0.6849468*	*0.014523*	*0.00096823*	*0.10682*	*1.467*	*2.8738*	0.0021 **
shape∼diet	0.7465781	0.003183	0.00079569	0.02356	1.1761	0.91903	0.1847
shape∼diet.50	0.7868153	0.008864	0.00080578	0.06542	1.1963	1.1981	0.1114
shape∼diet.matrix	0.7959216	0.007572	0.00084138	0.0558	1.2477	1.548	0.0601
shape∼habitat	0.7837622	0.007569	0.00084095	0.05589	1.2496	1.3506	0.0869
shape∼beak.function	0.7815233	0.003974	0.0007949	0.02936	1.1735	1.2622	0.1047
shape∼ trophic.level	0.7726645	0.001391	0.00046366	0.01028	0.6789	−0.87116	0.8101
shape∼trophic.niche	0.8438583	0.00732	0.00081328	0.05314	1.1847	1.4365	0.0733
shape∼primary.lifestyle	0.7775695	0.003394	0.00084839	0.02508	1.2539	1.4945	0.0686
shape∼log10(centroid size):Phy.group	*0.6771672*	*0.011333*	*0.00080947*	*0.08322*	*1.2615*	*1.7381*	0.0385 *
shape∼log10(centroid size):trophic.level	0.8191661	0.001946	0.00064858	0.01426	0.9511	0.01995	0.4910
shape∼log10(centroid size):trophic.niche	0.8425583	0.006827	0.00075850	0.04350	0.9770	−0.0669	0.5291
shape∼diet:habitat	0.7764997	0.012065	0.00054841	0.08916	0.7982	−1.39307	0.9167
shape∼diet.50:habitat	0.8325238	0.019816	0.00066054	0.14454	0.9731	−0.19079	0.5763
shape∼diet.50:trophic.level	0.798879	0.004648	0.00077466	0.03423	1.1459	0.54583	0.2834
shape∼diet.50:trophic.niche	0.8609643	0.004998	0.00071397	0.03596	1.0338	0.10159	0.4573
shape∼diet.50: log10(centroid size)	0.8552528	0.006703	0.00067027	0.04839	0.9800	0.19221	0.4266
shape∼diet.50: primary.lifestyle	0.7829707	0.014875	0.00082638	0.10985	1.2651	1.4555	0.0739 .
shape∼diet.50:Phy.group	0.55741	0.027277	0.00073723	0.19216	1.1325	1.0113	0.1580
shape∼diet.50: beak.function	0.8114272	0.012078	0.00063567	0.08869	0.9407	−0.05387	0.5184
shape∼beak.function: Phy.group	0.7281681	0.010208	0.00072913	0.07550	1.1210	0.67719	0.2482
shape∼trophic.level: trophic.niche	0.854422	0.003087	0.00077185	0.02230	1.1196	0.27648	0.3888
shape∼diet matrix:Phy.group	0.5711227	0.046277	0.00072308	0.32805	1.1328	1.1086	0.1375

### (c) Quadrate shape, avian phylogeny and allometry

The strongest correlation we found across Neornithes is between quadrate shape and phylogenetic group with *R^2^* of approximately 0.11, meaning that roughly 11% of total quadrate shape variation can be explained by the major phylogenetic lineage that the quadrate-bearer belongs to (table 1; electronic supplementary material, figure S24A). This comparatively significant relationship between clade and quadrate shape can be observed in the general distribution of clades in quadrate morphospace (electronic supplementary material, figure S13) and in the paradigmatic case of galloanserans that retain similar morphological attributes of the quadrate despite great variation in their feeding ecology (shaded area in figure 4*a*). Furthermore, we found a significant correlation between quadrate shape and size (quadrate allometry) which also varies depending on phylogenetic group (significant interaction log_10_ (centroid size):phylogenetic group in table 1 and electronic supplementary material, figure S24B). This is also the case for non-Telluraves (non-landbirds) which represent most of the species in our dataset (*n* = 137) with *R^2^* of approximately 0.02 (electronic supplementary material, table S4 and figure S24C), but this relationship is non-significant for Telluraves (electronic supplementary material, table S3; *N* = 63).

### (d) Quadrate shape, skull shape and internal integration

**Figure 5.**
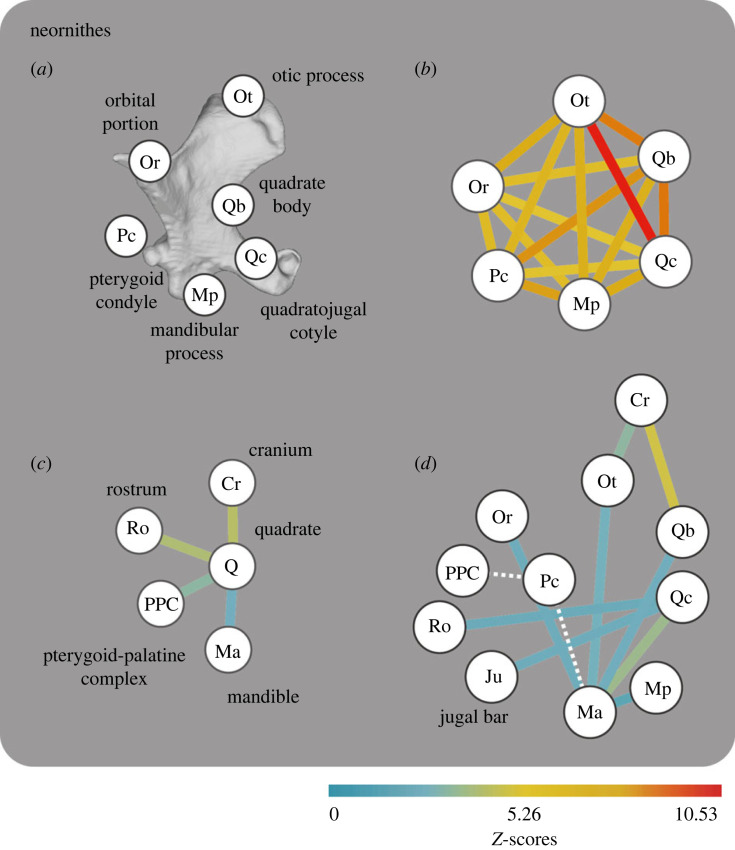
Evolutionary integration of the quadrate in Neornithes. (*a*) Abbreviation for portions of the quadrate illustrated in this figure. (*b*) Integration patterns among regions within the quadrate in Neornithes (*n* = 200). (*c*) Integration patterns between the quadrate and its neighbouring bones, and (*d*) between different areas/processes of the quadrate and neighbouring bones in Neornithes (*n* = 97). Dashed lines indicate non-significant relationships.

Quadrate shape strongly covaries with cranial (*R*^2^ = 0.6525, *Z* = 4.200631, *p* = 1 × 10^−04^) and mandibular shape (*R*^2^ = 0.5701, *Z* = 2.52311, *p* = 0.0053), but also with the shape of the rostral portion of the cranium (*R*^2^ = 0.6389, *Z* = 4.04526, *p* = 1 × 10^−04^) and the palatine-pterygoid complex (*R*^2^ = 0.5911, *Z* = 3.090950, *p* = 8 × 10^−04^) (PPC, mostly representing morphological variance of the palatine, see [46]; figure 5; electronic supplementary material, table S6). The shapes of individual regions of the quadrate also exhibit strong covariation with corresponding articular skeletal elements in the cranium and mandible; for instance, the orbital portion of the quadrate (orbital process + basiorbital fossa) and the mandible (*R*^2^ = 0.4368, *Z* = 2.369986, *p* = 0.0083), the otic process and the mandible (*R*^2^ = 0.4841, *Z* = 2.720581, *p* = 0.003), the otic process and the whole cranium (*R*^2^ = 0.508, *Z* = 3.215532, *p* = 4 × 10^−04^), the quadrate body and the mandible (*R*^2^ = 0.4982, *Z* = 2.490418, *p* = 0.0058), the quadrate body and the whole cranium (*R*^2^ = 0.6458, *Z* = 4.752419, *p* = 1 × 10^−04^), the quadratojugal cotyle and the mandible (*R*^2^ = 0.5507, *Z* = 3.572648, *p* = 2 × 10^−04^), the quadratojugal cotyle and the jugal bar (*R^2^* = 0.4147, *Z* = 2.742068, *p* = 0.0073), the quadratojugal cotyle and the rostrum (*R*^2^ = 0.369, *Z* = 2.080532, *p* = 0.0175) and the mandibular process and the mandible (*R*^2^ = 0.4779, *Z* = 1.706295, *p* = 0.0443). These relationships are generally invariant when analysed at lower phylogenetic scales within both Telluraves and non-Telluraves (electronic supplementary material, tables S7 and S8). However, we found no statistical differences between these relationships in Neornithes and both subclades (Telluraves and non-Telluraves) (electronic supplementary material, table S10). Shape covariation among all quadrate subregions is uniformly statistically significant and stronger than covariation between quadrate shape and other regions of the skull (figure 5; electronic supplementary material, table S6).

## 4. Discussion

The quadrate is a key component of the kinetic system of the skull in neornithine birds (figure 1) [18–21], and its functional relevance raises the possibility that strong selective pressures directly related to feeding and foraging ecology may have shaped its morphological evolution. However, our results challenge this assumption: the relationship between quadrate shape and feeding and foraging autecology in extant birds is generally either non-significant or weak (although variable, see below) at multiple phylogenetic scales (table 1; electronic supplementary material, table S3 and S4), even when accounting for potential confounding factors (electronic supplementary material, table S2). As such, the diversification of quadrate shape in Neornithes may not have been driven directly by selection for ecological optimality. Many-to-one (different shapes associated with the same ecology) and one-to-many (similar shapes associated with disparate ecologies) relationships between quadrate morphology and function are apparent from our results (figures 3 and 4). For instance, nectarivory, a highly specialized feeding ecology, is represented in our dataset by several distantly related species which all exhibit highly divergent quadrate shapes (i.e. many-to-one relationship of morphology and function; figure 4*b*), perhaps reflecting different mechanisms of nectar feeding associated with distinct phylogenetic histories [49], and/or that the morphology and function of other areas of the head like the tongue (including the hyoid skeleton) and/or the upper and lower jaws are simply more closely linked to nectar feeding than the quadrate is [50]. These results echo recent studies that have demonstrated that, at broad phylogenetic scales, avian evolution is characterized by complex relationships between skeletal morphology and ecology. For instance, dietary or other bill usage traits explain a relatively small portion of cranial shape variance across Neornithes [8,9], despite the fact that these form–function relationships are among the strongest observed across the entire avian skeleton [10]. Alternatively, it is possible that our ecological variables (i.e. diet and diet.50) do not comprehensively capture the ecological complexity of trophic niches in living birds, partly contributing to non-significant relationships emerging between avian quadrate shape variance and autecology under PGLS.

We found a much stronger, statistically significant link between avian quadrate geometry and phylogeny, with different avian lineages exhibiting distinctive quadrate shapes and clade-specific allometries (figure 4; relevant tables). For instance, while Anatidae (ducks, geese, swans) exhibit a wide spectrum of feeding ecologies ranging from piscivory (e.g. *Mergus*) to herbivory (e.g. *Sarkidiornis*), with beak morphologies reflecting this ecological disparity [11], they exhibit very similar quadrate morphologies overall (i.e. a one-to-many relationship of morphology to function; figure 4*a*; electronic supplementary material, figure S17 and table S5). Similarly, several morphological features of the quadrate represent autapomorphies diagnosing specific avian subclades. For instance, owls (Strigiformes) exhibit a unique caudomedial extension of the otic capitulum, and nightjars (Caprimulgidae) show a weakly developed orbital process. The latter is related to the loss of the *m. pseudotemporalis profundus* [51], which is thought to contribute to the enhanced capacity for large gape sizes characterizing the group [51], and to reorienting the lower jaw when closed [52]. Similarly, the mandibular process of Psittaciformes (parrots) is characterized by a single, deep medial condyle with a prominent articular surface, which enables the quadrate to slide rostrocaudally along its articular surface with the lower jaw to facilitate strong adduction [53]. These examples both illustrate a degree of clade-specific functional adaptation in the avian quadrate and underline the potential utility of quadrate morphology for phylogenetically placing fossil birds [54–57].

Our study focused on the evolutionary relationships between quadrate shape and ecology; however, our approach may have missed informative relationships between function and other aspects of quadrate form, such as size. The size of morphological structures has been shown to predict important aspects of avian ecology (e.g. sternum [58,59], cranium and mandible [10], and labyrinth [60,61]), and it stands to reason that relative quadrate dimensions may provide important, though as-yet unexplored insights into cranial function. The important role of the quadrate in avian cranial kinesis relates to its contribution to a mechanical four-bar linkage that drives elevation of the upper beak with respect to the cranium [62]. Although size variation (centroid size) of the avian quadrate is only weakly associated with shape variance (approx. 1%, table 1), the relative size of the quadrate (with respect to the other cranial elements), rather than geometric shape, may prove to be a more important aspect of quadrate morphology due to its influence on the mechanical advantage of the suspensorium and palate, a question that should be investigated in the future.

Our results investigating bird quadrates are strikingly different to those emerging from a recent study of squamate quadrate morphology, which showed that ecological habits explain a relatively large proportion of quadrate shape variance (20%), with little shape variance explained by phylogeny [63]. These divergent results in birds and squamates may have multiple explanations. For instance, there is some evidence that the bird quadrate may exhibit a comparatively stronger degree of evolutionary integration with the palate bones than the squamate quadrate [64,65] such that quadrate form in birds may be dictated to a greater extent by complex patterns of cranial structural integration rather than by selection for mechanical optimality for specific foraging modalities acting on individual cranial elements. Indeed, our analyses support this view: we found evidence for strong evolutionary integration between the shape of the quadrate and that of adjacent bony elements of the skull, such as the palate, at all phylogenetic scales (Neornithes, Telluraves and non-Telluraves), indicating that morphological evolution of the avian quadrate has been shaped to some degree by the evolution of the adjacent head skeleton. Nonetheless, our results also support the hypothesis that the quadrate has evolved as a coherent unit (i.e. an evolutionary module) within the avian cranial kinetic system as indicated by a stronger degree of covariation among regions within this element than between the quadrate and adjacent skeletal components (figure 5).

The integration between quadrate form and adjacent skeletal elements most likely reflects functional associations between components of the avian kinetic system. However, we found no evidence that these relationships vary at different phylogenetic levels (electronic supplementary material, table S10). This result might be affected by our uneven sampling across clades or our sampling within clades being insufficiently comprehensive. Increased sampling at lower phylogenetic scales will be needed in future studies to ascertain whether variation exists in patterns of covariation between quadrate morphology and neighbouring cranial elements among different avian lineages.

## 5. Conclusion

Collectively, our results underline the complexity of the evolution of the avian feeding apparatus and suggest that different lineages have frequently arrived at functionally comparable, yet morphologically divergent solutions. The three-dimensional complexity of avian quadrate geometry and the quadrate's tight functional integration within the suspensorium/palate region may underlie the tenuous relationship found between quadrate geometry and feeding autecology at a broad macroevolutionary scale, which appears to be far weaker than the relationships emerging from studies of craniofacial ecomorphology (e.g. [8,9]). Meanwhile, although our results suggest that quadrate shape variance is largely decoupled from feeding autecology or patterns of beak usage across the higher-level diversity of crown birds, the strongly integrated relationship between quadrate geometry (including the shape of its articular surfaces and muscle attachments) and adjacent bones within the skull suggest shape-related functional differences within the cranial kinetic system of different groups of birds. Future studies explicitly interrogating the biomechanical role of the quadrate within the avian cranial kinetic system should focus on additional aspects of quadrate morphometry (e.g. the width of the otic process and the quadrate body, the relative size of the orbital process to the quadrate body or the width of the mandibular process) to yield a deeper understanding of the relationship between quadrate morphology and beak function across extant bird phylogeny. Importantly, the significant association between quadrate shape and phylogeny highlights opportunities to incorporate quadrate geometry into investigations aiming to place fossil bird remains in a phylogenetic context using an explicitly quantitative framework. The frequency of well-preserved quadrates in the avian fossil record highlights the potential of this line of research for shedding light on the early evolutionary history of crown birds (e.g. [66–68]).

## Data Availability

The data that support the findings of this study are openly available in MorphoSource (https://www.morphosource.org/projects/000530134), reference no. 000530134. All data are presented in the electronic supplementary material [69]. Three-dimensional data will be made openly accessible from MorphoSource upon manuscript acceptance. Morphometric landmark coordinates, phylogenetic trees and code for our comparative phylogenetic analyses are provided at Zenodo (doi:10.5281/zenodo.10483479) [70].
